# Amino acid-based formula affects the gastrointestinal cytokine milieu of children with non-IgE mediated cow’s milk allergy

**DOI:** 10.1186/2045-7022-5-S3-P41

**Published:** 2015-03-30

**Authors:** Hannah E Jones, Anita Hartog, Holly Stephenson, Katja Brunner, Lucien F Harthoorn, Jane E Langford, Neil Shah, Mona Bajaj-Elliott, Keith Lindley

**Affiliations:** 1 Institute of Child Health, University College London, London, United Kingdom; 2 Utrecht Institute for Pharmaceutical Sciences, Utrecht University, Utrecht, The Netherlands; 3 Nutricia Research, Utrecht, The Netherlands; 4 Nutricia Advanced Medical Nutrition, Nutricia Research, Utrecht, The Netherlands; 5 Nutricia Advanced Medical Nutrition, Liverpool, United Kingdom; 6 Great Ormond Street Hospital, London, United Kingdom

## Introduction

Food Allergy (FA) presents a significant health and economic burden in the western world. Children with non-IgE mediated cow's milk allergy (CMA) are being increasingly seen in clinic. Diagnosis is largely based on delayed onset of symptoms, primarily affecting the gastrointestinal (GI) mucosa. Treatment involves an elimination diet supplemented with amino acid-based formula (AAF) which in some children results in effective symptom relief. To understand the beneficial effects of AAF at the molecular level, herein we characterized the GI cytokine milieu *ex vivo* from children with or without AAF in their elimination diets.

## Methods

35 pediatric patients (≤ 5 years) undergoing a diagnostic colonoscopy for their GI symptoms were recruited. Biopsies were collected from the ascending colon. Biopsies were cultured for 24 hours with media only (baseline) or 250µg/ml AAF (Neocate). Supernatants and biopsies were collected and immune mediators quantified by real-time PCR, ELISA and multiplex cytokine assay.

## Results

Biopsy supernatants from children with AAF in their diet showed a significant reduction in baseline levels of pro-inflammatory cytokines TNF-α and IL-6 compared to those without AAF. Interestingly, addition of Neocate *ex vivo* directly reduced IL-1β, IL-6 and TNF-α in biopsies from children without AAF in their diet but not those with AAF. Neocate treatment also led to a significant reduction in epithelial-derived TSLP and IL-33. Similarly, Th2 (IL-4, IL-13) and Treg (IL-10) associated cytokines were significantly reduced in AAF diet group compared to the non-AAF group.

## Discussion

These data provide evidence for the mode of action of AAF on the GI mucosa. The reduction of inflammatory cytokines *ex vivo* supports the hypothesis that a specific AAF (i.e. Neocate) may have direct anti-inflammatory effects, thus offering one explanation as to how these formula help in non-IgE CMA. Detailed analyses of how AAF affects inflammatory cells are underway [1].

## Consent

Written informed consent was obtained from the parent or guardian of the patients for publication of this abstract and any accompanying images. A copy of the written consent is available for review by the Editor of this journal.
